# Adherence to the EAT-Lancet diet and its relation with food insecurity and income in a Brazilian population-based sample

**DOI:** 10.1590/0102-311XEN247222

**Published:** 2023-12-22

**Authors:** Mariana Alves Ferreira, Alexsandro Macedo Silva, Dirce Maria Lobo Marchioni, Eduardo De Carli

**Affiliations:** 1 Faculdade de Saúde Pública, Universidade de São Paulo, São Paulo, Brasil.

**Keywords:** Food Consumption, Food Security, Poverty, Consumo Alimentar, Segurança Alimentar, Pobreza, Consumo Alimentario, Seguridad Alimentaria, Pobreza

## Abstract

This study aimed to investigate the relation of adherence to the planetary diet with food and nutrition security status and per capita household income in a study with a representative sample of the Brazilian population. Among the data from the 2017-2018 *Brazilian Household Budgets Survey* (POF), the inequality indicators selected for the analysis were data on per capita household income and food and nutrition security. We also considered data on the individual food consumption of 46,164 Brazilians aged ≥ 10 years, obtained through 24-hour dietary recalls, in the *National Food Survey*, conducted with the POF 2017-2018. The *Planetary Health Diet Index* (PHDI) was used to measure adherence to the planetary diet. Sociodemographic data were expressed as frequency (%), with analysis of the mean and 95% confidence interval (95%CI) of the PHDI score. The relation of food and nutrition security and income with the PHDI score was tested in multiple linear regression models. The calculations were performed in the Stata software, adopting a 5% significance. Lower PHDI means were observed among food insecure individuals, male, < 20 years old, mixed-race and indigenous, with income < 0.5 minimum wage, residing in rural areas and in the North and Northeast regions. In the multiple linear regression, food insecurity was inversely related to PHDI score (ꞵ = -0.56; 95%CI: -1.06; -0.06), with the lowest scores associated with severe food insecurity (β = -1.31; 95%CI: -2.19; -0.55). Income categories were not independently associated to PHDI score (p-trend = 0.900). Therefore, food insecurity has been shown to negatively affect Brazilians’ adherence to the planetary diet.

## Resumo

O objetivo deste estudo foi investigar a relação entre a adesão à dieta planetária com a situação de segurança alimentar e nutricional e renda familiar *per capita*, utilizando amostra representativa da população brasileira. Entre os dados da *Pesquisa de Orçamentos Familiares* (POF) de 2017-2018, os indicadores de desigualdade selecionados para a análise foram as informações sobre renda familiar *per capita* e segurança alimentar e nutricional. Também foram considerados dados de consumo alimentar individual de 46.164 brasileiros com idade ≥ 10 anos, obtidos por meio de recordatórios alimentares de 24 horas, no *Inquérito Nacional de Alimentação*, conduzido junto à POF 2017-2018. O *Índice de Dieta Planetária* (PHDI) foi empregado para mensurar a adesão à dieta planetária. Dados sociodemográficos foram expressos como frequência (%), com análise da média e intervalo de 95% de confiança (IC95%) do escore do PHDI. A relação entre segurança alimentar e nutricional e renda com o escore do PHDI foi testada em modelos de regressão linear múltipla. Os cálculos foram executados no software Stata, adotando uma significância de 5%. Menores médias do PHDI foram observadas entre indivíduos em insegurança alimentar, do sexo masculino, < 20 anos, pardos e indígenas, com renda < 0,5 salário mínimo, domiciliados na zona rural e das regiões Norte e Nordeste. Na regressão linear múltipla, a insegurança alimentar foi inversamente relacionada ao escore do PHDI (ꞵ = -0,56; IC95%: -1,06; -0,06), sendo as menores pontuações associadas à insegurança alimentar grave (β = -1,31; IC95%: -2,19; -0,55). As categorias de renda não foram independentemente associadas com o escore PHDI (p de tendência = 0,900). Portanto, a insegurança alimentar demonstrou afetar negativamente a adesão dos brasileiros à dieta planetária.

## Resumen

El objetivo de este estudio fue investigar la relación entre la adherencia a la dieta planetaria con la situación de seguridad alimentaria y nutricional y el ingreso familiar per cápita en un estudio con una muestra representativa de la población brasileña. Entre los datos de la *Encuesta de Presupuestos Familiares* (POF) 2017-2018, los indicadores de desigualdad seleccionados para el análisis fueron la información sobre el ingreso familiar per cápita y la seguridad alimentaria y nutricional. También se utilizaron los datos de consumo alimentario individual de 46.164 brasileños ≥ 10 años, obtenidos mediante registros de alimentos de 24 horas, en la *Encuesta Nacional Alimentaria*, realizada con POF 2017-2018. Se utilizó el *Índice de Dieta Planetaria* (PHDI) para medir la adherencia a la dieta planetaria. Los datos sociodemográficos se expresaron como frecuencia (%), con análisis de la media e intervalo de 95% de confianza (IC95%) de la puntuación del PHDI. La relación entre el seguridad alimentaria y nutricional y los ingresos con la puntuación del PHDI se probó en modelos de regresión lineal múltiple. Los cálculos se realizaron en el software Stata, con el nivel de significación del 5%. Se observaron medias más bajas del PHDI entre individuos con inseguridad alimentaria, hombres, < 20 años, pardos e indígenas, con ingresos < 0,5 salario mínimo, residentes en zonas rurales y en las regiones Norte y Nordeste de Brasil. En la regresión lineal múltiple, la inseguridad alimentaria se relacionó inversamente con la puntuación del PHDI (ꞵ = -0,56; IC95%: -1,06; -0,06), y las puntuaciones más bajas estaban asociadas con la inseguridad alimentaria grave (β = -1,31; IC95%: -2,19; -0,55). Las categorías de ingresos no se asociaron de forma independiente con la puntuación PHDI (p de tendencia = 0,900). Por lo tanto, la inseguridad alimentaria afecta negativamente la adherencia de los brasileños a la dieta planetaria.

## Introduction

The United Nations’ (UN) Sustainable Development Goals (SDGs) demonstrated the importance of taking action to combat inequalities, hunger, poverty, and environmental impacts, considering the need to promote a more sustainable agricultural system to guarantee human and planetary health ^1^. In line with the SDGs, the EAT-Lancet Commission on Food, Planet and Health released in 2019 the proposal for a world reference guide, establishing intervals for the intake of selected food groups, known as planetary diet ^2^.

The EAT-Lancet Commission describes in its report that a planetary diet is composed of plant diversity, with small portions of animal foods, favoring unsaturated fats, whole grains and limiting added sugars ^2^. Recently, Cacau et al. ^3^ developed the *Planetary Health Diet Index* (PHDI), whose construction was based on the healthy and sustainable reference diet model proposed by the EAT-Lancet Commission, and was validated to measure adherence to the planetary diet.

However, data on the populations’ accessibility to this planetary diet model are still little explored ^4^, especially among social vulnerable populations. Food insecurity represents the limitation of full and permanent access to food, with hunger being associated with its most severe form ^5^ ^,^ ^6^. According to the most recent report of the United Nations Food and Agriculture Organization (FAO), published in 2022 ^7^, about 924 million people in the world were exposed to the most severe form of food insecurity. Previous studies have shown that income also has an important association with access to adequate food ^8^ ^,^ ^9^. Thus, this study aimed to research the relation of adherence to the planetary diet with food security status and per capita household income.

## Methods

### Brazilian Household Budgets Survey

The *Brazilian Household Budgets Survey* (POF, acronym in Portuguese) is a nationwide survey, carried out by sampling, and its research unit is the household. It aims to collect information such as per capita household income, household expenses, living conditions and consumption habits of Brazilian households ^10^. The POF data collection takes place over 12 months, and information is obtained via interviews conducted in private households for nine consecutive days.

As in the previous edition ^11^, the POF 2017-2018 carried out the *Brazilian National Food Survey* (INA, acronym in Portuguese), whose objective was to collect data on individual food consumption and obtain dietary estimates for the total population, as well as stratified for sex, age groups, monthly household income, urban or rural settings and macroregions. In the 2017-2018 edition, the INA covered 20,112 randomly selected households, which corresponds to a subsample of 34.7% of the total 57,920 households investigated in the POF, totaling information on the food consumption of 46,164 individuals aged 10 years or older. In this same edition, and for the first time in this survey, data on food and nutrition security status were collected. The POF 2017-2018 microdata were obtained from the official Brazilian Institute of Geography and Statistics (IBGE, acronym in Portuguese) website ^10^.

### Food and nutrition security

The *Brazilian Food Insecurity Scale* (EBIA, acronym in Portuguese) was used in the sixth block of POF to obtain data on food and nutrition security ^11^. Pérez-Escamilla et al. ^6^ developed the EBIA by adapting the scale of the United States Department of Agriculture (USDA). The questionnaire has 14 questions, with yes or no questions, and each positive answer represents 1 point. The food security category is determined by scores equal to 0. Food insecurity has three degrees of severity, and the scores that determine each depend on the absence or presence of people aged under 18 years at the household, being divided into mild (1-5 points in the presence of people aged < 18 years and 1-3 points in the absence of people aged < 18 years), moderate (6-9 points in the presence of people aged < 18 years and 4-5 points in the absence of people aged < 18 years), and severe (10-14 points in the presence of people aged < 18 years and 6-8 points in the absence of people aged < 18 years) ^11^.

### Per capita family income

Disposable income is included among the data of the expenditure profile of Brazilian families and the income of the population was collected through information on total income, which includes nonmonetary income. Disregarding equity variation, monetary income covers all forms of monetary gain during the 12-month period prior to the interview, and nonmonetary income takes into account gains from goods and services acquired in a nonmonetary manner (e.g., donation, withdrawal from the business, exchange, or own production) ^10^. Disposable income results from the sum of the total monetary and nonmonetary income of the consumption unit, divided by the total number of residents, characterizing per capita family income ^10^. Per capita household income data were used, categorizing individuals according to the availability of 2018 minimum wages (MW), which was BRL 954.00 ^10^, into the following categories: up to 0.5, 0.5-1, 1-2, and > 2.

### Brazilian National Food Survey

Individual food consumption data were obtained through two 24-hour dietary recalls (24hR), collected from the interviewed households, on nonconsecutive days ^10^. The interview was developed following a plan structured in sequential stages based on the multiple passage method ^12^, with the aid of a tablet application program. For each food, the app provides information on the home units of measurements to allow the estimation of the quantity consumed. In this edition, the database had a total of 1,832 registered foods. The table of teferred measures for food consumed in Brazil - developed in the POF 2008-2009 ^13^, revised and updated in the POF 2017-2018 - helped to estimate the quantities of consumption in grams or milliliters of each food and beverage ^10^. Only the first 24hR collected was considered for this study, and this food consumption was representative for weekdays and weekends, in all months of the year.

### Planetary Health Diet Index

The PHDI was used to assess adherence to the EAT-Lancet diet. To meet the recommendations proposed by the EAT-Lancet Commission, Cacau et al. ^3^ defined 16 components, which were grouped into four categories: adequacy components (nuts and peanuts, legumes, fruits, vegetables, and whole cereals); optimum components (eggs, dairy products, fish and seafood, tubers and potatoes, and vegetable oils); ratio components (dark green vegetables/total ratio and red and orange vegetables/total ratio); and (4) moderation components (red meat, chickens and substitutes, animal fats, and added sugars).

In the 24hRs, fresh or minimally processed foods (fruits, cooked vegetables) were identified, as well as preparations with multiple ingredients, which require the dismemberment of their ingredients to be classified into the components of the PHDI, including culinary preparations based on a main ingredient (e.g., foods with sauce, added oil, butter or salt), mixed preparations (e.g., *feijoada*, cakes) and industrialized processed products (e.g., snacks, soft drinks). The ingredients of culinary or mixed preparations were broken down from homemade recipe standards contained in national references ^14^ ^,^ ^15^ ^,^ ^16^. As described by Cacau et al. ^3^, industrialized products based on a main component (e.g., maize starch salty chips) had their energetic fractionation based on their main ingredients and its content of added sugars or total fat. Following the example of a salty chip, the energy percentage of total fats is assumed to be the contribution of the vegetable oil fraction in that food. After deducing the total fats from the chips, the contribution of the refined grain group (maize starch) is assumed to be the remainder of the energy value of this food.

Following the dismemberment of culinary or mixed recipes and industrialized processed products, the ingredients within the components considered by the diet proposed by the EAT-Lancet Commission ^2^ were classified as in Cacau et al. ^3^. The score of each component of the index is based on its energy contribution to total intake (i.e., total of foods that were classified in one of the components ÷ total of foods included in the PHDI * 100). According to their type (adequacy, optimum, ratio or moderation), each food group composing the diet had its score calculated depending on how its intake values are close or far from the cut-off points (maximum score) and/or limits (minimum score) established in the reference diet. Adequacy, optimum and moderation components score up to 10 points, while ratio components score up to 5 points. The final index score is gradual, ranging from 0 to 150 points. Details on the PHDI can be found in the original publication that describes its development and validation according to the level of consumption relative to the total energy value ^3^.

### Statistical analysis

The set of sociodemographic variables available and used in the analyses were: sex (male and female), years of education (≤ 8, ≥ 9 and ≤ 11, and ≥ 12), age group in years (< 20, 20-30, 31-45, 46-59, and > 60), region of the country (North, Northeast, Southeast, South, and Central-West), home area (urban or rural), self-declared ethnicity/skin color (white, black, brown, yellow, and indigenous), and nutritional status (low weight, normal weight, overweight, and obesity). Body mass index (BMI - kg/m^2^) of the individuals was calculated according to the self-reported weight and height in the POF data collection, to classify it according to the categories of nutritional status among adolescents (< 20 years) - through the z-score - and adults (20-30, 31-45, and 46-59 years old), considering the cut-off points established by the World Health Organization (WHO) ^17^, and those of Lipschitz among older adults (> 60 years) ^18^.

Descriptive data were expressed as frequency (%), means and their respective 95% confidence intervals (95%CI). The percentage of food insecurity and low income in Brazil was expressed in each Federative Unit (UF, acronym in Portuguese), as well as the maximum percentage of the PHDI score, relative to the total of 150 points. The PHDI score was analyzed according to sociodemographic variables. The score, the percentage of energy contribution to the daily total, and the consumption in grams per day (g/d) of each food group that compose the PHDI were also evaluated according to extreme categories of per capita household income and food and nutrition security. In descriptive analyses, statistical differences between means were identified in the absence of intersection of their 95%CIs.

By multiple linear regression models, the PHDI score was related to the variation in per capita household income (reference: < 0.5 minimum wage) and to the food and nutrition insecurity status (reference: food security). A stepwise forward procedure was used to include adjustment variables in the multiple regression models, retaining those that were significantly associated with the PHDI score. Three models were presented: the first related to the univariate analysis; the second, adjusted for sex (reference: male), age group (reference: < 20 years) and self-declared ethnicity/skin color (reference: white); and the third added with the other modifiable sociodemographic variables: years of education (reference: up to 8), home area (reference: urban), region (reference: North), BMI (kg/m^2^), and total energy value of the diet (kcal/day). Also, possible interactions of sociodemographic and nutritional covariates with per capita household income and food and nutrition insecurity status were tested. The final models were tested for multicollinearity, using variance inflation factor (VIF), and for residual normality, using graphical analysis of histograms and Q-Q plots. Statistical analysis was performed using Stata 14.0 program (https://www.stata.com), considering the complexity of the sample and the expansion factors, applying the survey command in all calculations. In all multiple linear regression analyses, a 5% significance level was adopted.

## Results

The prevalence of food insecurity among Brazilians aged ≥ 10 years in 2017-2018 was 40.9%. As shown in Table 1, compared to individuals in food security, those with some degree of food insecurity had around 1.4 points lower mean in the PHDI (46.4 vs. 45.0, respectively). The statistical differences between food security and food insecurity statuses occurred in both sexes and areas of residence, being especially evident among individuals in the age group > 60 years, self-declared as white and mixed-race, living in the Northeast Region, individuals with up to 11 years of education, with per capita household income of up to 0.5 minimum wage, and with nutritional status classified as normal weight or overweight.

**Table 1:** *Planetary Health Diet Index* (PHDI) score means according to sociodemographic characteristics stratified by food security status. Brazil, 2017-2018.

Characteristics	PHDI score					
	Food security			Food insecurity		
	%	Mean	95%CI	%	Mean	95%CI
Total	59.0	46.4	46.1; 46.7	40.9	45.0	44.7; 45.4
Sex						
Female	30.3	47.0	46.6; 47.4	21.8	45.3	44.9; 45.6
Male	28.7	45.8	45.5; 46.2	19.1	44.8	44.3; 45.2
Age group (years)						
< 20	8.4	43.9	43.3; 44.5	9.4	43.5	42.9; 44.1
20-30	10.6	44.8	44.0; 45.5	7.9	43.8	43.2; 44.5
31-45	14.7	46.2	45.7; 46.7	11.0	45.3	44.8; 45.8
46-59	12.8	47.5	46.9; 48.0	7.4	46.4	45.8; 47.0
> 60	12.3	48.7	48.2; 49.3	5.2	46.9	46.1; 47.8
Per capita household income (minimum wages)						
Up to 0.5	3.8	45.3	44.5; 46.1	10.2	43.9	43.3; 44.4
0.5-1	11.5	46.0	45.4; 46.6	14.5	44.6	44.0; 45.2
1-2	22.0	46.6	46.1; 47.0	12.5	46.0	45.2; 46.7
> 2	21.6	46.7	46.1; 47.3	3.6	46.6	45.6; 47.7
Self-declared ethnicity/skin color						
White	30.2	46.5	46.1; 46.9	12.8	45.1	44.5; 45.7
Black	5.4	46.5	45.7; 47.3	05.3	45.8	44.9; 46.7
Brown	22.5	46.2	45.7; 46.7	22.3	44.8	44.4; 45.3
Indigenous	0.2	40.9	37.6; 44.3	0.2	41.5	39.1; 44.0
Yellow	0.6	50.0	46.5; 54.7	0.1	45.1	42.0; 48.1
Education (years)						
≤ 8	21.3	46.5	46.1; 46.8	20.5	44.9	44.6; 45.4
≥ 9 and ≤ 11	9.1	46.6	45.9; 47.1	7.6	44.9	44.2; 45.6
≥ 12	28.5	46.3	45.8; 46.8	12.7	45.2	44.6; 45.8
Region						
North	3.0	45.7	44.7; 46.7	5.2	44.4	43.6; 45.1
Northeast	12.3	44.0	43.6; 44.5	14.6	42.4	42.0; 42.8
Southeast	27.8	47.5	46.9; 48.0	14.8	47.2	46.5; 48.0
Central-West	4.8	48.4	47.6; 49.1	2.8	48.3	47.3; 49.4
South	11.0	45.9	45.3; 46.4	3.4	44.9	43.5; 46.3
Home area						
Urban	51.9	46.5	46.1; 46.8	33.5	45.2	44.8; 45.7
Rural	7.0	46.2	45.6; 46.7	7.4	44.1	43.5; 44.7
Nutritional status						
Low weight	2.9	47.1	45.8; 48.4	2.1	45.4	44.3; 46.5
Normal weight	26.9	46.2	45.8; 46.6	19.6	44.7	44.2; 45.2
Overweight	19.9	46.5	45.9; 46.9	12.8	45.2	44.8; 45.7
Obesity	9.2	46.8	46.2; 47.5	6.3	45.6	44.9; 46.2

95%CI%: 95% confidence interval.

**Figure 1:**
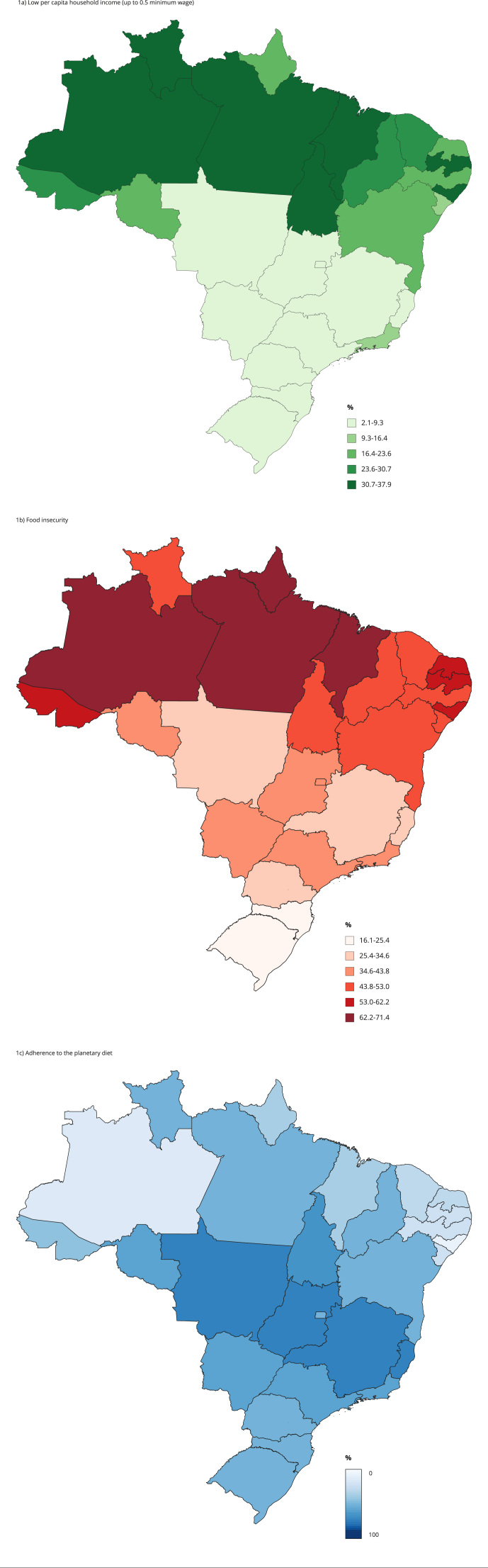
Distribution of the low per capita household income, food insecurity status, and percentage of adherence to the planetary diet in the Brazilian Federative Units.

In the map of the distribution of the PHDI score percentage among the Brazilian UFs, it can be observed that Amazonas, Pará, Acre, Amapá, Maranhão, Rio Grande do Norte, and Alagoas, presented a high concentration of individuals with per capita household income of up to 0.5 minimum wage (Figure 1a), as well as the highest prevalence of food insecurity (Figure 1b). The same seven UFs had the lowest means for adherence to the planetary diet, corresponding to 27.3%, 30.2%, 30.4%, 29.3%, 28.8%, 28.2%, and 25.8% of the total of 150 points of the PHDI, respectively (Figure 1c).

One of the multiple linear regression models presented in Table 2 relates the PHDI score and the food and nutrition security status. It was observed that, in particular, severe food insecurity was the most significantly associated with lower scores, with its cases presenting a 1.31 points lower mean PHDI score than that estimated for those in food security (β = -1.31; 95%CI: -2.12; -0.50).

**Table 2:** Multiple linear regression of the association of the *Planetary Health Diet Index* (PHDI) score with per capita household income classes and food insecurity degrees.

	PHDI score					
	Univariate model		Model 1 *		Final model 2 **	
	β	95%CI	β	95%CI	β	95%CI
Food security	Reference		Reference		Reference	
Food insecurity	-1.39	-1.85; -0.92	-0.96	-1.43; -0.49	-0.51	-1.00; -0.02
Food security	Reference		Reference		Reference	
Mild food insecurity	-1.07	-1.61; -0.53	-0.65	-1.19; -0.11	-0.36	-0.91; 0.19
Moderate food insecurity	-1.75	-2.57; -0.93	-1.32	-2.14; -0.50	-0.62	-1.43; 0.18
Severe food insecurity	-2.62	- 3.45; -1.79	-2.10	-2.94; -1.26	-1.31	-2.12; -0.50
p-trend	< 0.001		< 0.001		0.003	
Income categories (minimum wages)						
Up to 0.5	Reference		Reference		Reference	
0.5-1	0.96	0.34; 1.58	0.64	0.01; 1.27	0.09	-0.51; 0.70
1-2	2.09	1.46; 2.73	1.34	0.69; 1.99	0.19	-0.46; 0.85
> 2	2.42	1.70; 3.15	1.36	0.58; 2.14	-0.06	-0.88; 0.75
p-trend	< 0.001		< 0.001		0.903	

95%CI%: 95% confidence interval.

* Adjusted for sex, self-declared ethnicity/skin color and age group;

** Adjusted for education, sex, self-declared ethnicity/skin color, age group, home area, region, body mass index, total energy intake.

As shown in Table 2, the income categories showed a direct association with the PHDI score in univariate models (p-trend < 0.001) and adjusted for sex, self-declared ethnicity/skin color and age group (p-trend < 0.001), but lost significance when considering the other sociodemographic variables (p trend = 0.903). There was no evidence of significant interactions between food security status or per capita household income with the sociodemographic and nutritional variables tested on the PHDI score.

To better understand the factors related to lower adherence to the planetary diet, mean consumption in g/day, mean percentage of caloric contribution relative to the daily total, and mean score of the food groups that compose the PHDI were evaluated according to the extreme categories of per capita household income (up to 0.5 and > 2 minimum wages), and food and nutrition security (food security and severe food insecurity) (Table 3).

**Table 3:** Consumption in grams, daily energy percentage and mean score of the *Planetary Health Diet Index* (PHDI) components, stratified by the extremes of per capita household income and food and nutrition security status.

PHDI components	Per capita household income (minimum wages)				Food security		Severe food insecurity	
	> 2		Up to 0.5					
	Mean	95%CI	Mean	95%CI	Mean	95%CI	Mean	95%CI
Nuts and peanuts								
Consumption (g)	1.73	1.43; 2.02	0.94	0.70; 1.18	1.19	1.040; 1.340	1.46	0.79; 2.13
Daily energy percentage	0.50	0.41; 0.56	0.24	0.18; 0.30	0.31	0.28; 0.35	0.32	0.19; 0.45
Mean score	0.34	0.29; 0.40	0.19	0.14; 0.23	0.23	0.20; 0.25	0.22	0.14; 0.30
Legumes								
Consumption (g)	165.20	159.30; 171.00	179.10	169.20; 189.00	154.00	149.30; 158.60	154.50	141.40; 167.50
Daily energy percentage	5.03	4.80; 7.18	8.12	7.72; 8.51	6.44	6.26; 6.61	7.62	6.97; 8.27
Mean score	3.85	3.70; 4.01	5.54	5.32; 5.76	4.75	4.64; 4.87	5.00	4.65; 5.34
Fruits								
Consumption (g)	219.70	208.40; 230.90	123.30	113.20; 133.50	186.62	180.30; 192.90	115.20	102.00; 128.40
Daily energy percentage	7.14	6.84; 7.45	3.97	3.66; 4.30	6.02	5.58; 6.20	4.08	3.60; 4.56
Mean score	6.09	5.90; 6.30	3.77	3.55; 4.00	5.37	5.25; 5.49	3.60	3.30; 3.92
Total vegetables								
Consumption (g)	114.60	110.40; 118.70	64.30	61.00; 67.60	101.90	99.20; 104.50	60.00	54.90; 64.60
Daily energy percentage	2.53	2.40; 2.67	1.94	1.81; 2.07	2.37	2.30; 2.45	1.90	1.71; 2.08
Mean score	5.83	5.70; 5.97	4.70	4.54; 4.85	5.54	5.46; 5.62	4.60	4.36; 4.83
Whole grains								
Consumption (g)	27.00	26.00; 28.00	24.10	23.05; 25.3	8.82	7.90; 9.74	3.24	1.02; 5.45
Daily energy percentage	1.52	1.33; 1.71	0.28	0.21; 0.35	1.01	0.91; 1.11	0.37	0.19; 0.55
Mean score	0.46	0.40; 052	0.08	0.06; 0.10	0.30	0.27; 0.33	0.11	0.06; 0.17
Eggs								
Consumption (g)	17.00	15.90; 18.10	15.4	13.90; 16.80	16.20	15.60; 17.00	18.20	15.60; 20.80
Daily energy percentage	1.47	1.36; 1.60	1.57	1.41; 1.73	1.43	1.34; 1.56	2.12	1.76; 2.50
Mean score	0.92	0.84; 1.00	0.43	0.37; 0.50	0.74	0.70; 0.80	0.54	0.44; 0.63
Fish and seafood								
Consumption (g)	15.70	13.00; 18.40	32.60	28.00; 37.30	14.90	13.40; 16.60	39.90	31.20; 48.60
Daily energy percentage	1.19	0.97; 1.41	2.36	2.04; 2.70	1.07	0.96; 1.20	2.77	2.20; 3.36
Mean score	0.13	0.09; 0.17	0.10	0.07; 0.13	0.09	0.07; 0.11	0.09	0.05; 0.14
Potatoes and tubers								
Consumption (g)	50.00	46.50; 53.50	38.20	34.50; 42.00	45.20	42.80; 47.50	45.20	38.70; 51.70
Daily energy percentage	3.83	3.60; 4.08	4.85	4.40; 5.31	3.62	3.45; 3.80	6.15	5.30; 7.00
Mean score	0.95	0.83; 1.07	0.51	0.42; 0.60	0.83	0.77; 0.90	0.52	0.38; 0.67
Dairy products								
Consumption (g)	146.10	139.60; 152.60	67.80	62.90; 72.60	126.60	122.80; 130.30	60.00	52.20; 67.80
Daily energy percentage	9.11	8.75; 9.47	3.47	3.24; 3.71	7.30	7.10; 7.51	3.03	2.73; 3.32
Mean score	2.66	2.54; 2.80	2.28	2.13; 2.43	2.65	2.57; 2.72	1.94	1.75; 2.14
Plant oils								
Consumption (g)	27.00	26.00; 27.90	24.10	23.00; 25.30	27.30	26.70; 27.90	22.70	21.30; 24.10
Daily energy percentage	12.20	12.00; 12.60	11.30	10.90; 11.70	12.20	12.00; 12.40	11.20	10.70; 11.80
Mean score	5.45	5.34; 5.55	5.60	5.44; 5.74	5.60	5.54; 5.67	5.56	5.35; 5.77
Red meat								
Consumption (g)	98.00	93.80; 102.30	89.20	83.00; 95.50	99.4	96.80; 102.10	72.60	65.10; 80.10
Daily energy percentage	12.60	12.00; 13.10	12.10	11.30; 13.00	12.7	12.40; 13.00	10.40	9.42; 11.4
Mean score	2.54	2.37; 2.72	3.65	3.40; 3.90	2.77	2.66; 2.88	4.35	3.94; 4.76
Chicken and substitutes								
Consumption (g)	45.50	42.30; 48.80	53.50	49.10; 58.00	50.00	47.70; 52.50	51.00	45.00; 57.00
Daily energy percentage	4.90	4.47; 5.33	6.51	6.01; 7.01	5.46	5.20; 5.73	6.61	5.83; 7.39
Mean score	5.17	4.96; 5.37	4.47	4.22; 4.73	5.08	4.95; 5.21	4.17	3.82; 4.53
Animal fat								
Consumption (g)	4.09	3.62; 4.55	2.30	1.80; 2.82	3.60	3.30; 3.90	1.93	1.40; 2.45
Daily energy percentage	1.41	1.24; 1.60	0.84	0.61; 1.06	1.24	1.14; 1.34	0.74	0.56; 0.93
Mean score	7.70	8.20; 8.45	9.02	8.84; 9.19	8.15	8.05; 8.26	9.06	8.87; 9.25
Added sugars								
Consumption (g)	46.80	44.90; 48.60	37.30	35.50; 39.10	46.30	45.10; 47.40	33.90	31.20; 36.60
Daily energy percentage	9.95	9.65; 10.20	8.64	8.26; 9.02	9.97	9.77; 10.1	8.34	7.77; 8.92
Mean score	1.90	1.75; 2.02	2.53	2.34; 2.73	1.97	1.89; 2.06	2.81	2.53; 3.09
Dark-green vegetables								
Consumption (g)	7.06	6.22; 7.90	2.05	1.22; 2.90	5.18	4.72; 5.65	1.97	1.31; 2.62
Daily energy percentage	5.46	4.87; 6.05	2.80	1.54; 4.05	4.26	3.94; 4.57	2.50	1.87; 3.14
Mean score	0.60	0.52; 0.66	0.29	0.22; 0.35	0.47	0.43; 0.50	0.27	0.21; 0.34
Red and orange vegetables								
Consumption (g)	47.80	45.20; 50.40	20.80	18.90; 22.70	40.50	39.00; 42.00	18.40	16.00; 20.90
Daily energy percentage	27.10	26.10; 28.20	12.70	11.70; 13.60	23.10	22.50; 23.80	11.90	10.60; 13.20
Mean score	2.04	2.00; 2.16	1.05	0.98; 1.13	1.81	1.77; 1.86	0.98	0.88; 1.09

95%CI%: 95% confidence interval.

Individuals in the lowest quarter of income, compared to the highest, had a higher mean consumption, in grams, of legumes and fish and seafood, and lower than other food groups. The highest mean for fish intake and lowest consumption of other food groups was also found among individuals at the most severe food insecurity in relation to those in food security, with the exception of legumes, potatoes, eggs, and nuts. The consumption of red meat by individuals with per capita household income of up to 0.5 minimum wage was 9% lower compared to those with > 2 minimum wages. On the other hand, individuals in severe food insecurity consumed 26.9% less red meat compared to those who were in food security. There was no evidence of differences in chicken and substitute consumption between the extreme food and nutrition security categories.

At both extremes of income and food security, the PHDI scores followed the observed differences for consumption in grams of most adequacy, moderation, and ratio food components. Among the optimum components, low scores resulted from consumption above the average recommended value for dairy products among individuals with > 2 minimum wages and in food security, and from consumption above the average recommended value for fish and seafood among individuals with < 0.5 minimum wage and in severe food insecurity. In addition, low consumption of nuts and peanuts, and whole grains provided worse scores among the extreme categories of per capita household income and food and nutrition security. In the case of total vegetables and fruits, individuals with income > 2 minimum wages and in food security had better scores in relation to individuals with income up to 0.5 minimum wage and in severe food insecurity, with a difference in consumption in grams of 43.8% and 38.2%, respectively. For the consumption of legumes, there was no evidence of differences between the extremes of food and nutrition security (Table 3).

## Discussion

This study investigated the relation of adherence to the planetary diet with food insecurity and income, applying the PHDI ^3^ to individual food consumption data from a study with a representative sample of the Brazilian population aged ≥ 10 years. It was observed that food insecurity was negatively related to the PHDI score; however, per capita household income was not a determinant for adherence to the PHDI, regardless of other sociodemographic characteristics.

The dietary patterns found in this sample showed that the Brazilian population in general presented low consumption of adequacy components such as fruits, vegetables, nuts and peanuts, and whole grains. Legume consumption followed a different pattern, with the highest score among the adequacy components, which may be related to the high prevalence of bean consumption, a traditional staple in Brazilian eating habits, as highlighted in the POF 2017-2018 individual consumption analysis report ^9^. Regarding red meat, although lower consumption in grams was observed in individuals with income up to 0.5 minimum wage and in severe food insecurity, it was beyond that recommended by the EAT-Lancet Commission, reflecting in low scores in the PHDI score, regardless of the categories of per capita household income and food and nutrition security. The prevalence of consumption of legumes, such as beans, and red meat could be explained by greater cultural acceptance, since these components are strongly associated with the local food culture ^19^ ^,^ ^20^.

Verly Junior et al. ^21^ conducted a study using the same sample as our analysis, showing that to achieve recommendations for a healthy diet at the lowest possible cost, low-income families could still face high spending on food to meet the consumption of fruits and vegetables. In addition, a study by Ricardo & Claro ^22^, with data from the POF 2008-2009, related the cost of food with the energy density of the diet of Brazilians, and the results identified higher prices associated with foods such as fruits, vegetables and legumes, demonstrating that income is an important factor to access a healthier diet and lower calorie density ^22^. According to our findings, individuals with per capita household income up to 0.5 minimum wage consumed significantly lower amounts of fruits and vegetables compared to people with income > 2 minimum wages, reinforcing that income can be a limiting factor for a diet with foods considered healthier and more sustainable.

A global analysis conducted by Hirvonen et al. ^9^ demonstrated through the investigation of the cost of the foods that make up the planetary diet that low-income populations could have difficulties in meeting the recommendations of the EAT-Lancet Commission. It has already been described in a previous study that the planetary diet adherence score means for Brazilian individuals in the lowest income quartile were lower, compared to those in the highest income quartile ^23^. However, according to our findings, per capita household income was not associated with the PHDI score, regardless of other sociodemographic characteristics. It is worth mentioning, in this sense, recognized limitations of average per capita income measures, since they tend to be underestimated, especially in the richest households ^24^, and is not able to reflect variations between household units with differential requirements attributable to the composition of residents in different life cycles ^25^. In addition, individuals are subject to different contexts, in addition to those related to the availability of income, which constitute a challenge for adherence to the planetary diet, since there are differences in the cost of living ^26^, prices and availability of food ^5^ ^,^ ^27^ between the regions of the country, being factors that could lead to local inequalities in the accessibility to a quality diet.

On the other hand, food insecurity was inversely related to adherence to the planetary diet, with lower PHDI scores as its severity increased, regardless of other sociodemographic characteristics. Similarly to income, we observed great inequality in the distribution of food insecurity among the federated units of Brazil. In locations with high rates of food insecurity, the lowest PHDI score percentages were also observed, suggesting an important role of this phenomenon for the worsening of dietary quality in the Brazilian population.

Marchioni et al. ^23^ demonstrated in their study that the average PHDI scores were lower among younger people, living in rural areas, living in the North and Northeast regions, and with the lowest incomes. The POF 2017-2018 report on food security indicates that these same groups are the most susceptible to food insecurity ^11^. The present analysis also observed that the consumption in grams of important food groups for compliance with the recommendations of the EAT-Lancet Commission, such as fruits, total vegetables and whole grains, were significantly lower when individuals are in the most severe form of food insecurity, reinforcing that food insecurity is a phenomenon that negatively impacts adherence to the planetary diet.

Low income is described as one of the main determinants of food insecurity. However, the availability and price of food, with influences arising from the food system, as well as the cost of other essential basic needs at the local level, are also determinants for food insecurity, being factors that go beyond the income available to ensure full access to food ^23^ ^,^ ^24^ ^,^ ^25^ ^,^ ^26^ ^,^ ^27^ ^,^ ^28^ ^,^ ^29^. In Brazil, programs geared to promoting food and nutrition security were effective in combating food insecurity and poverty ^30^. However, policies promoting food and nutrition security lost space on the Brazilian political agenda as a result of a crisis that began around 2014, which intensified food insecurity in the country in 2017-2018, affecting the quality of life and nutrition of the population ^31^.

It is recognized that the food and nutrition security data collected through the EBIA constitute a limitation of the study, since they refer to the household level and not necessarily to an individual level, as prioritized in this work. However, the EBIA questionnaire enables obtaining data on different dimensions of the food insecurity phenomenon, with different cutoff points for households with the presence or absence of people aged under 18 years, thus being able to express how each degree of food insecurity can affect individuals in the household ^6^.

It is also important to recognize that underreporting of food consumption is a common limitation in population-based studies that use surveys such as 24hR, and it is not possible to predict which foods specifically will be subject to this bias. On the other hand, the 24hR is the most used tool in population-based studies, as it has the lowest associated measurement error ^32^, with the POF 2017-2018 data collection having followed strict methodological standards, in order to favor the quality of the information obtained ^10^ ^,^ ^12^. Furthermore, in order to ensure greater accuracy of the analysis and considering that the use of a 24hR measure is described as an appropriate method for studies interested in describing and comparing group-level food consumption means ^33^ ^,^ ^34^, we used the first 24hR, since it is recognized as the measure that is the least subject to biases related to underreporting of energy intake ^35^ ^,^ ^36^.

To the best of our knowledge, this is the first study to relate adherence to the diet proposed by the EAT-Lancet Commission with food insecurity and income in a representative sample of the Brazilian population. Among the strengths of the study, it is worth mentioning the use of data from the most recent national food survey ^10^, with representation of all regions of the country and urban and rural household situations. Additionally, the PHDI was used to assess adherence to the planetary diet because it is a validated index that has already performed well in differentiating diets both in terms of nutritional aspects and environmental impacts ^3^ ^,^ ^23^, being, therefore, an important tool for assessing food consumption from the perspective of the recommendations of the EAT-Lancet Commission. To this end, an extensive work of classification of the planetary diet components was carried out following methodological standards ^3^ ^,^ ^14^ ^,^ ^15^ ^,^ ^16^, in order to guarantee the reliability and validity of the results.

## Conclusion

In the context of Brazilian eating habits, food insecurity, but not income, negatively affected adherence to the planetary diet. This reinforces that, in order to achieve sustainable goals, it is important that populations are guaranteed the human right of access to adequate food, with a political agenda that prioritizes combating inequalities and strengthens the promotion of sustainable and fair food systems to provide adequate food for all.
